# Leisure sedentary time is differentially associated with hypertension, diabetes mellitus, and hyperlipidemia depending on occupation

**DOI:** 10.1186/s12889-017-4192-0

**Published:** 2017-03-23

**Authors:** Man Sup Lim, Bumjung Park, Il Gyu Kong, Songyong Sim, So Young Kim, Jin-Hwan Kim, Hyo Geun Choi

**Affiliations:** 1 Department of General Surgery, Hallym University Sacred Heart Hospital, 22, Gwanpyeong-ro 170, Dongan-gu, Anyang-si, Gyeonggi-do 14068 Republic of Korea; 2 Department of Otorhinolaryngology-Head & Neck Surgery, Hallym University Sacred Heart Hospital, 22, Gwanpyeong-ro 170, Dongan-gu, Anyang-si, Gyeonggi-do 14068 Republic of Korea; 3 Department of Statistics, Hallym University, 1, Hallym-ro, Chunchon-si, Kwangwon-do 24252 Republic of Korea; 4 Department of Otorhinolaryngology-Head & Neck Surgery, Bundang Cha Hospital, 59, Yatop-ro, Bundang-gu, Gyeonggi-do, 13496 Republic of Korea; 5 Department of Otorhinolaryngology-Head & Neck Surgery, Hallym University Kangnam Sacred Heart Hospital, 1, Shingil-ro, Youngdongpo-gu, Seoul, 07441 Republic of Korea

**Keywords:** Sedentary lifestyle, Leisure activities, Hypertension, Diabetes mellitus, Hyperlipidemia, Occupations, Work

## Abstract

**Background:**

Sedentary behavior is considered an independent cause of cardio-metabolic diseases, regardless of physical activity level and obesity. Few studies have reported the association between leisure sedentary time and cardio-vascular diseases in terms of occupation.

**Methods:**

We performed a cross-sectional study using data from the Korean Community Health Survey (KCHS) for 240,086 participants assessed in 2011 and 2013. Occupation was categorized into four groups: farmer or fisherman, laborer, and soldier (Group I); service worker, salesperson, technician, mechanic, production worker, and engineer (Group II); manager, expert, specialist, and clerk (Group III); and unemployed (Group IV). Leisure sedentary time was divided into five groups: 0 h, 1 h, 2 h, 3 h, and 4+ h. The association between leisure sedentary time on weekdays and hypertension/diabetes mellitus/hyperlipidemia for different occupations was analyzed using simple and multiple logistic regression analyses with complex sampling.

**Results:**

In Groups I, II and III, no length of sedentary time was associated with hypertension, and only 3 h or 4+ h of sedentary time was associated with diabetes mellitus and hyperlipidemia. Group IV showed a significant association with hypertension and diabetes mellitus for the 2 h, 3 h, and 4+ h sedentary times.

**Conclusions:**

The unemployed are more susceptible than other occupation groups to cardio-metabolic diseases when leisure time is sedentary.

**Electronic supplementary material:**

The online version of this article (doi:10.1186/s12889-017-4192-0) contains supplementary material, which is available to authorized users.

## Background

Sedentary behavior is generally defined as any waking behavior characterized by an energy expenditure ≤1.5 metabolic equivalent test score (METs) while in a sitting or reclining position [1]. Sedentary time is associated with all-cause mortality [2], diabetes [3], cancers [4, 5], cardiovascular diseases [6], and obesity [7]. It is considered an independent cause of cardio-metabolic diseases, regardless of physical activity and obesity in many studies [8–10].

For adults, sedentary time could be divided into workplace sedentary time, including commute time, and leisure sedentary time. Adults usually spend approximately 1/3 of their weekday time (approximately 1/2 of their weekday awake time) working. It is expected that the physical activity or sedentary behavior patterns of each occupation would be different. Because of the relatively large proportion of working hours in a 24-h day, the differences in working behaviors might affect cardio-metabolic disease differently. However, it is difficult to accurately measure the differences in physical activity and sedentary behavior in the workplace [11], and few studies have evaluated the differences in physical activity and sedentary time among occupations [12–14]. Therefore, it is difficult to evaluate the association between sedentary time in the workplace and health. Moreover, despite knowing the effects of workplace sedentary time on health and having the intention to change behavior, changing the pattern or activity in the workplace is very difficult for most people.

In contrast to time spent in the workplace, the use of leisure time can be changed relatively easily through individual effort, and leisure sedentary time is more predominant than workplace sedentary time (~60%) [14]. Therefore, in this study, we focused on the use of leisure time for sedentary behavior and its association with cardio-metabolic diseases such as hypertension, diabetes mellitus, and hyperlipidemia. We hypothesize that leisure time sedentary behavior is associated differently with cardio-metabolic diseases according to the different occupation groups. We calculated the adjusted odds ratios (AORs) of leisure sedentary time on weekdays for hypertension, diabetes mellitus, and hyperlipidemia for different occupations. To our knowledge, no study has reported the differences in association of sedentary time with cardio-vascular diseases according to occupation.

## Methods

### Study population and data collection

This study was approved by the Institutional Review Board of Korea Centers for Disease Control and Prevention (IRB No. 2011-05CON-04-C and 2013-06EXP-01-3C). Written informed consent was obtained from all participants prior to the survey.

This study is a cross-sectional study using data from the Korean Community Health Survey (KCHS). The KCHS conducted in 2011 and 2013 was analyzed. The data were collected by the Centers for Disease Control and Prevention of Korea. The survey was administered through face-to-face, paper-assisted personal interviews between trained interviewers and respondents. The sample size for the KCHS was 900 subjects in each of 253 community units, including 16 metropolitan cities and provinces. The KCHS used a two-stage sampling process. In the first stage, a sample area (*tong/ban/ri*) was selected as a primary sample unit, which was selected according to the number of households in the area using a probability proportional to the sampling method. In the second stage, the number of households in the selected sample *tong/ban/ri* was identified to create a household directory. Sample households were selected using systematic sampling methods. This process was used to ensure that the sample units were representative of the entire population [15]. For the sample to be statistically representative of the population, the data collected from the survey were weighted by statisticians based on the sample design [16]. According to KCHS, 9.2% of the participants refused to complete the survey.

**Fig. 1:**
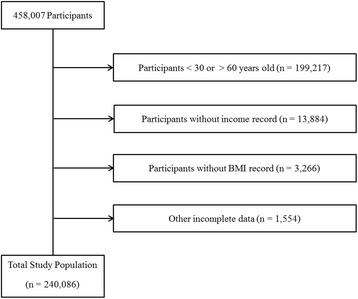
A schematic illustration of participant selection in the present study Among a total of 458,007 participants, those aged under 30 or over 60 years or with incomplete survey data were excluded from this study. The data for the 240,086 participants from whom complete data were obtained were analyzed.

Of a total of 458,007 participants, we excluded the following participants from this study: participants under 30 years old or over 60 years old (199,217 participants); participants who did not complete the income record (13,884 participants); participants who did not report height or weight (3266 participants); and participants who had incomplete data for educational level, occupation, smoking, alcohol consumption history, sleep hours, stress level, physical activity, sedentary time, hypertension, diabetes mellitus, and hyperlipidemia (1554 participants). Ultimately, 240,086 participants (112,559 male; 127,527 female) were included in this study (Fig. 1).

### Survey

To measure the moderate-intensity physical activity, the participants were asked “How often do you do light or moderate leisure time physical activities for at least 10 min that cause only light sweating or a slight-to-moderate increase in breathing or heart rate?”. Participants answered for both the frequency and duration of moderate-intensity physical activities. To measure the vigorous-intensity physical activity, the participants were asked “How often do you do vigorous leisure time physical activities for at least 10 min that cause heavy sweating or large increases in breathing or heat rate?”. Participants answered for both the frequency and duration of vigorous physical activities, as well. One minute of vigorous activity was counted as two minutes of moderate activity. Then, the sums of vigorous and moderate physical activity were combined and measured as moderate-intensity physical activity (MPA). This MPA was divided into three groups: 0 min/week (inactive); 1–149 min/week (insufficiently active); ≥ 150 min/week (sufficiently active) [17].

Occupation was classified into ten standard Korean occupations: manager; expert; specialist; clerk; service worker; salesperson; farmer or fisherman; technician, mechanic, production worker, or engineer; laborer; and soldier. Unemployed participants comprised an eleventh group. Then, it was categorized into four groups according to their possible physical activities: farmer or fisherman, laborer, and soldier (Group I); service worker, salesperson, technician, mechanic, production worker, and engineer (Group II); manager, expert, specialist, and clerk (Group III); and unemployed (Group IV). Although we did not measure each occupation group’s physical activity level or sedentary time during working hours, our grouping of theses occupations is consistent with common sense and other study results [12, 14, 18]. For example, in general, office workers are expected to have less physical activity than manual workers in the workplace [12, 19].

Using the methods recommended by the Organization for Economic Cooperation and Development [20] (i.e., dividing household income by the square root of the number of household members), monthly income was divided into lowest, low-middle, upper-middle, and highest quartiles.

To evaluate the sedentary time according to occupation, the participants less than 30 years of age or over 60 years of age were excluded from this study. These exclusion limits were based on the theory that these age groups could be unemployed due to college enrollment, job-seeking activities or retirement. Participants under 110 cm or 30 kg were excluded from this study. Using the World Health Organization’s [21] international classifications for adult underweight, overweight and obesity according to body mass index (BMI, kg/m^2^), four BMI groups were established: underweight, < 18.5; healthy, ≥ 18.5, < 25; overweight ≥25, < 30; obese, ≥ 30.

To explore the influence of educational level, uneducated participants, and those who had graduated only from elementary or middle schools, were assigned to the “low” education group; high school graduates comprised the “middle” group, and junior college graduates, college and graduate school graduates formed the “high” group.

Smoking status was divided into three groups: non-smoker; past smoker; and current smoker. The past smokers who had quit smoking less than one year prior to the study were included in the current smoker group. Alcohol consumption was divided into the following four categories: None; ≤ 1 time a month; 2–4 times a month; ≥ 2 times a week. Amount of sleep was divided into four groups: 3–5 h per day; 6 h per day; 7 h per day; and ≥8 h per day. Participants who sleep less than three hours per day were excluded from this study (0.2% of the participants were excluded). The participants were asked if they usually feel stress, and stress levels were divided into the following four groups: no stress; some stress; moderate stress; and severe stress.

To measure sedentary time, the question asked was, “How much time did you spend per day sitting to watch TV, play games, use the internet, or do other things, during your leisure time on weekdays during the past week?” The participants chose from five possible answers: < 1 h (0 h); ≥ 1 h, < 2 h (1 h); ≥ 2 h, < 3 h (2 h); ≥ 3 h, < 4 h (3 h); ≥ 4 h (4+ h). Weekend sedentary time was also surveyed. Because both weekday and weekend sedentary times were surveyed as categorical variables, it was not possible to combine them into one category. Therefore, we chose only weekday sedentary time for this study.

The participants were questioned about their histories of hypertension, diabetes mellitus, and hyperlipidemia, and those who reported a history of any of these diseases, as diagnosed by a medical doctor, were recorded as positive. The questionnaire was described in the Additional file 1.

### Statistical analyses

Differences in mean age among occupation groups were compared using linear regression analysis with a complex sampling test. The rate differences of gender, income level, BMI group, educational level, occupation, alcohol consumption, smoking history, sleep hours, stress level, hypertension, diabetes mellitus, and hyperlipidemia history were compared by using the Chi-square test with Rao-Scott correction.

To identify associations between sedentary time and hypertension, diabetes mellitus, and hyperlipidemia, simple and multiple logistic regression analyses with complex sampling were used. In the multiple logistic regression analysis, age, sex, income, obesity, education, alcohol, smoking, stress, physical activity, sleep, and leisure sedentary time were adjusted as the confounders. For the subgroup analysis according to the occupation groups (Groups I to IV), multiple logistic regression analysis with complex sampling was used. Two-tailed analyses were conducted, and *P*-values lower than 0.05 were considered to indicate significance. The AOR and 95% confidence interval (CI) for hypertension, diabetes mellitus, and hyperlipidemia were calculated. All results are presented as weighted values. The results were analyzed statistically using SPSS ver. 21.0 (IBM, Armonk, NY, USA).

## Results

The total 240,086 participants were distributed across the occupation Groups I, II, III, and IV as follows: 44,116 (18.4%), 75,912 (31.6%), 63,213 (26.3%), 56,845 (23.7%), respectively. Group I (farmer or fisherman, laborer, and soldier) showed the highest mean age, relatively lower income and educational level, and higher MPA. Group II (service worker, salesperson, technician, mechanic, production worker, and engineer) exhibited middle educational level and the highest smoking rate. Group III (manager, expert, specialist, and clerk) displayed the highest income and educational level, and relatively less sleep time. Group IV (unemployed) showed a higher female rate, the lowest smoking and alcohol consumption, relatively lower MPA, and longer sleep time (Table 1).

**Table 1:** General characteristic of participants according to occupation group

	Total	Group I	Group II	Group III	Group IV	*P*-value
Number						
N	240,086	44,116	75,912	63,213	56,845	
%	100	18.4	31.6	26.3	23.7	
Age (y)	44.4	48.5	45.0	41.8	45.1	<0.001*
Sex (%)						<0.001*
Male	51.0	52.8	66.2	62.9	13.8	
Female	49.0	47.2	33.8	37.1	86.2	
Income (%)						<0.001*
Lowest	6.8	13.0	4.9	2.5	12.0	
Low-middle	23.4	34.9	26.2	12.7	28.0	
Upper-middle	33.4	31.7	37.1	31.2	31.9	
Highest	36.5	20.5	31.8	53.6	28.2	
Obesity (%)						<0.001*
Underweight	3.8	2.4	2.4	3.8	6.3	
Healthy	70.3	70.3	68.7	69.2	74.1	
Overweight	23.6	25.1	26.4	24.8	17.5	
Obese	2.3	2.1	2.5	2.3	2.1	
Education (%)						<0.001*
Low	14.5	38.4	15.7	1.3	18.7	
Middle	39.1	46.2	54.0	19.7	40.6	
High	46.4	15.4	30.3	79.0	40.7	
Alcohol (%)						<0.001*
None	20.2	23.1	15.2	14.5	33.2	
< 1 time a month	27.0	25.0	22.4	26.2	35.1	
2–4 times a month	26.3	22.0	27.1	31.5	20.6	
≥ 2 times a week	26.5	29.9	35.3	27.8	11.1	
Smoking (%)						<0.001*
None	57.0	53.9	42.6	52.2	84.7	
Past	15.7	15.1	19.5	19.2	6.3	
Current	27.2	31.0	37.9	28.6	9.0	
Stress (%)						<0.001*
Little	13.4	17.0	12.0	10.3	17.8	
Mild	57.4	57.3	57.4	57.3	57.6	
Moderate	25.5	22.8	26.9	28.4	21.2	
Severe	3.6	2.9	3.7	4.0	3.4	
Physical activity (%)						<0.001*
0 m	52.6	49.8	51.9	48.6	60.0	
1–149 m	9.5	6.1	8.3	12.5	8.9	
≥ 150 m	37.9	44.1	39.8	38.9	31.1	
Sleep (%)						<0.001*
3–5 h	14.4	16.8	15.0	13.4	13.7	
6 h	33.4	32.4	34.8	37.4	26.7	
7 h	34.1	32.0	33.8	35.6	33.4	
≥ 8 h	18.1	18.8	16.3	13.6	26.1	
Leisure sedentary time (%)						<0.001*
< 1 h	20.5	18.9	22.0	24.2	14.6	
≥ 1 h, <2 h	34.3	35.1	36.1	37.5	27.1	
≥ 2 h, <3 h	23.5	25.2	23.9	21.2	24.9	
≥ 3 h, <4 h	10.4	11.8	9.7	8.0	14.0	
≥ 4 h	11.3	9.0	8.3	9.1	19.4	
Hypertension (%)						<0.001*
No	87.6	83.1	87.0	90.1	87.2	
Yes	12.4	16.9	13.0	9.9	12.8	
Diabetes mellitus (%)						<0.001*
No	95.4	93.8	95.2	96.7	94.7	
Yes	4.6	6.2	4.8	3.3	5.3	
Hyperlipidemia (%)						<0.001*
No	90.0	88.9	90.2	90.6	89.5	
Yes	10.0	11.1	9.8	9.4	10.5	

*Significance at *P* < 0.05

The ORs of sedentary time for hypertension, diabetes mellitus, and hyperlipidemia showed significance in both simple and multiple logistic regression with complex sampling with a dose-response relationship (Each, *P* < 0.001; Table 2). These results were consistent in other statistical model (Additional file 2).

**Table 2:** Odds ratios of sedentary time for hypertension, diabetes mellitus, and hyperlipidemia using simple and multiple logistic regression analyses with complex sampling

Sedentary time (h)	Hypertension		Diabetes mellitus		Hyperlipidemia	
	AOR (95% CI)	*P* Value	AOR (95% CI)	*P* Value	AOR (95% CI)	*P* Value
Simple regression		<0.001*		<0.001*		<0.001*
< 1 h	1		1		1	
≥ 1 h, <2 h	1.06 (0.99–1.15)		1.07 (1.02–1.12)		1.00 (0.95–1.06)	
≥ 2 h, <3 h	1.18 (1.09–1.28)		1.15 (1.10–1.21)		1.08 (1.02–1.14)	
≥ 3 h, <4 h	1.47 (1.34–1.61)		1.29 (1.22–1.37)		1.26 (1.18–1.34)	
≥ 4 h	1.76 (1.62–1.92)		1.45 (1.37–1.54)		1.38 (1.29–1.47)	
Multiple regression		<0.001*		<0.001*		<0.001*
< 1 h	1		1		1	
≥ 1 h, <2 h	1.02 (0.95–1.11)		1.02 (0.97–1.07)		0.98 (0.93–1.03)	
≥ 2 h, <3 h	1.09 (1.00–1.18)		1.06 (1.00–1.11)		1.03 (0.98–1.10)	
≥ 3 h, <4 h	1.23 (1.12–1.36)		1.09 (1.03–1.17)		1.15 (1.08–1.23)	
≥ 4 h	1.40 (1.28–1.53)		1.22 (1.15–1.30)		1.24 (1.17–1.33)	

*Significance at *P* < 0.05

Independent factors in the simple regression: leisure sedentary time

Independent factors in the multiple regression: Age, sex, income, obesity, education, alcohol, smoking, stress, physical activity, sleep, and leisure sedentary time

We calculated the AORs of sedentary time for hypertension, diabetes mellitus, and hyperlipidemia in each occupation group. In Group I, compared with 0 h of sedentary time, 1 h, 2 h, 3 h, and 4+ h no significance differences with regard to hypertension; 3 h (AOR = 1.22, 95% CI = 1.00–1.49) and 4+ h (AOR = 1.34, 95% CI = 1.09–1.64) of sedentary time was associated with diabetes mellitus (*P* = 0.027), and 4+ h (AOR = 1.30, 95% CI = 1.10–1.52) was linked to hyperlipidemia (*P* = 0.001). In Group II, compared with 0 h sedentary time, only 4+ h (AOR = 1.38, 95% CI = 1.18–1.62) was related to diabetes mellitus (*P* < 0.001). In Group III, compared with 0 h sedentary time, 3 h (AOR = 1.46, 95% CI = 1.17–1.82) was related to diabetes mellitus (*P* = 0.002) and 3 h (AOR = 1.17, 95% CI = 1.02–1.34) and 4+ h (AOR = 1.33, 95% CI = 1.17–1.51) were associated with hyperlipidemia (*P* < 0.001). In Group IV, compared with 0 h sedentary time, 2 h (AOR = 1.20, 95% CI = 1.06–1.36), 3 h (AOR = 1.21, 95% CI =1.05–1.38) and 4+ h (AOR =1.35, 95% CI = 1.19–1.53) were linked to hypertension (*P* < 0.001); 2 h (AOR = 1.20, 95% CI =1.00–1.44), 3 h (AOR = 1.24, 95% CI =1.02–1.51), and 4+ h (AOR =1.38, 95% CI = 1.16–1.65) were related to diabetes mellitus (*P* = 0.007); 3 h (AOR = 1.20, 95% CI = 1.04–1.38), and 4+ h (AOR = 1.18, 95% CI =1.04–1.35) correlated with hyperlipidemia (*P* < 0.001; Table 3).

**Table 3:** Subgroup analysis of adjusted odds ratios of sedentary time for hypertension, diabetes mellitus, and hyperlipidemia according to the occupation groups using multiple logistic regression analyses with complex sampling

Occupation group	Hypertension		Diabetes mellitus		Hyperlipidemia	
Sedentary time (h)	AOR (95% CI)	*P* value	AOR (95% CI)	*P* value	AOR (95% CI)	*P* value
Group I		0.039*		0.027*		0.001*
< 1 h	1		1		1	
≥ 1 h, <2 h	0.95 (0.86–1.06)		1.04 (0.89–1.23)		0.96 (0.83–1.10)	
≥ 2 h, <3 h	1.00 (0.90–1.13)		1.10 (0.92–1.30)		1.00 (0.87–1.14)	
≥ 3 h, <4 h	1.14 (0.99–1.31)		1.22 (1.00–1.49)		1.13 (0.96–1.32)	
≥ 4 h	1.09 (0.95–1.26)		1.34 (1.09–1.64)		1.30 (1.10–1.52)	
Group II		0.062		<0.001*		0.522
< 1 h	1		1		1	
≥ 1 h, <2 h	1.01 (0.94–1.09)		0.97 (0.86–1.09)		0.98 (0.90–1.07)	
≥ 2 h, <3 h	0.98 (0.90–1.07)		0.98 (0.86–1.12)		0.99 (0.90–1.08)	
≥ 3 h, <4 h	1.01 (0.91–1.12)		1.06 (0.90–1.24)		1.05 (0.93–1.18)	
≥ 4 h	1.15 (1.03–1.29)		1.38 (1.18–1.62)		1.07 (0.95–1.21)	
Group III		0.150		0.002*		<0.001*
< 1 h	1		1		1	
≥ 1 h, <2 h	1.00 (0.91–1.09)		0.97 (0.83–1.13)		1.01 (0.92–1.11)	
≥ 2 h, <3 h	1.09 (0.98–1.21)		1.11 (0.93–1.31)		1.10 (0.99–1.22)	
≥ 3 h, <4 h	1.06 (0.92–1.22)		1.46 (1.17–1.82)		1.17 (1.02–1.34)	
≥ 4 h	1.12 (0.98–1.28)		1.16 (0.93–1.44)		1.33 (1.17–1.51)	
Group IV		<0.001*		0.007*		<0.001*
< 1 h	1		1		1	
≥ 1 h, <2 h	1.12 (0.99–1.27)		1.18 (0.99–1.42)		0.90 (0.79–1.03)	
≥ 2 h, <3 h	1.20 (1.06–1.36)		1.20 (1.00–1.44)		1.01 (0.89–1.16)	
≥ 3 h, <4 h	1.21 (1.05–1.38)		1.24 (1.02–1.51)		1.20 (1.04–1.38)	
≥ 4 h	1.35 (1.19–1.53)		1.38 (1.16–1.65)		1.18 (1.04–1.35)	

*Significance at *P* < 0.05

Independent factors in the multiple regression: Age, sex, income, obesity, education, alcohol, smoking, stress, physical activity, sleep, and leisure sedentary time

## Discussion

In agreement with many previous studies [22–26], our study confirmed that sedentary time was associated with hypertension, diabetes mellitus, and hyperlipidemia. Leisure sedentary time was independently associated with these diseases with dose-response relationships when adjusting for MPA, sleep time, and other demographic characteristics (Table 2). Going one step further, this study revealed that the associations between sedentary time and cardio-metabolic diseases were different according to occupation groups (Table 3). To our knowledge, this study is the first to analyze these associations in each different occupation group.

Groups I and II, which were both physically active working groups, did not show significance for hypertension with regard to sedentary leisure time, and revealed significance for 3 h and 4+ h (Group I, hypertension) and 4+ h alone (Group I, hyperlipidemia; Group II, diabetes and hyperlipidemia) leisure sedentary time. Warren TY, et al. reported that sedentary time did not increase the risk of cardiovascular disease in physically active participants, whereas it increased this risk for those who were inactive [26]; however, Wijndaele K, et al. stated that sedentary time increased the risk of mortality regardless of physical activity [27]. We believe that long sedentary times (3 h or 4+ h) could increase the risk of cardio-metabolic diseases even in physically active working groups, whereas these effects would not occur in short sedentary times (1 h and 2 h).

Group III is the most highly educated group in this study, and most sedentary jobs require higher educational levels [12]. Therefore, we hypothesized that Group III was a more sedentary working group than Group I or II. In this group (Group III), only 4+ h of leisure sedentary time was associated with diabetes mellitus and 3 h and 4+ h with hyperlipidemia. This result is similar to the results of Groups I and II. Group III did not show the prominent associations between sedentary time and cardio-metabolic diseases that we expected. We believe that the unadjusted factors in this study such as healthy dietary habits, lifestyle, and the environment of highly educated and wealthy people might affect the results of Group III.

Group IV, which comprised the unemployed population, showed a significant association between cardio-metabolic diseases even with 2 h leisure sedentary time. In other studies, unemployed persons showed longer sedentary time and lower physical activity than any other occupation groups [12, 28]. The unemployed might be more susceptible to a short leisure sedentary time because of their lower physical activity. This result might be explained because they have longer non-leisure sedentary time, and adding leisure sedentary time could further increase the risk of cardio-metabolic disease. Because the majority of this group (86.2%) were women, we performed an additional analysis for women (Additional file 3). In this analysis, unemployed women showed a significant relationship between leisure sedentary time and cardio-metabolic diseases, while employed women did not.

This study has various advantages. It compared the association between leisure sedentary time and cardio-metabolic diseases among different occupation groups within a large population-based survey for the first time. Sampling was weighted by statisticians to reflect the mother population. We adjusted for MPA, sleep time, and obesity, which could act as confounding factors [29, 30], when evaluating the association between sedentary time and cardio-metabolic diseases.

Despite these advantages, this study has several limitations. First, this study used self-reported measurement of sedentary behavior and MPA. Self-reported measurements could be inaccurate due to recall bias. However, objective measurement using an accelerometer also has bias owing to incomplete data and measurement errors [31]. In some reports [32], self-reported sedentary behavior showed a better association with cardio-metabolic risks than objective measurement. Second, we used only weekday leisure sedentary time in that we surveyed sedentary time as a categorical variable. The exclusion of leisure sedentary time on weekends may have acted as a hidden bias. Third, we grouped eleven occupations into four groups without exact measurement of the physical activity and sedentary time in the workplace, relying on other study results. Physical activity and sedentary time might differ among participants with the same occupation. The absence of this information could have distorted the results. Fourth, measurement outcomes were based on medical reports. Because participants who are more likely to attend medical visits are more likely to have more significant disease histories, this outcome measurement method could have resulted in a bias. Fifth, we did not have access to the participants’ work records. Finally, the study was subject to the same limitations that affect all cross-sectional studies, including possible reverse causality; therefore, our calculated ORs should be interpreted with caution.

## Conclusions

This study indicates that the unemployed could be more susceptible to cardio-metabolic diseases when using leisure time for sedentary behavior than other occupation groups. Therefore, we should pay more attention to the leisure time use of the unemployed.

## Additional files

Additional file 1: The Questionnaire of the study. The surveyed questionnaire was described. (DOCX 16 kb)

Additional file 2: Odds ratios of sedentary time for hypertension, diabetes mellitus, and hyperlipidemia using multiple logistic regression analyses with complex sampling. In other statistical model, the odd ratios of sedentary time were described. (DOCX 20 kb)

Additional file 3: Subgroup analysis of adjusted odds ratios of sedentary time for hypertension, diabetes mellitus, and hyperlipidemia according to the occupation groups in female using multiple logistic regression analyses with complex sampling. This data shows the odd ratios of female groups. (DOCX 21 kb)

## Abbreviations

AORs: Adjusted odds ratios
BMI: Body mass index
CI: Confidence interval
KCHS: The Korean Community Health Survey
METs: Metabolic equivalent test score
MPA: Moderate-intensity physical activity
