# Supplementary material for: Leisure sedentary time is differentially associated with hypertension, diabetes mellitus, and hyperlipidemia depending on occupation
Source: BMC Public Health. 2017 Mar 23;17:278. doi: 10.1186/s12889-017-4192-0 (PMC5364658; doi:10.1186/s12889-017-4192-0)
Supplement: Supplementary file 1 — The Questionnaire of the study. The surveyed questionnaire was described. (DOCX 16 kb) [file 12889_2017_4192_MOESM1_ESM.docx]

Supplement 1. The questionnaire of the study

**Age** ___ year

**Sex** male/female

**Weight** ___kg

**Height** ___cm

**Income** What is your annual income in the recent one year? ___won

**Education** What is your final graduation of education? ① Elementary school ② Middle schools ③ High school ④ Junior college ⑤ College ⑥ Graduate school

**The moderate-intensity physical activity** How often do you do light or moderate leisure time physical activities for at least 10 min that cause only light sweating or a slight-to-moderate increase in breathing or heart rate? ___day ___hours ___ minutes

**The vigorous-intensity physical activity** How often do you do vigorous leisure time physical activities for at least 10 min that cause heavy sweating or large increases in breathing or heat rate? ___day ___hours ___ minutes

**Occupation** What is your job? ① manager ② expert or specialist ③ clerk ④ service worker ⑤ salesperson ⑥ farmer or fisherman ⑦ technician ⑧ mechanic, production worker, or engineer. ⑨ laborer ⑩ soldier ⑪ unemployed

**Smoking** Have you ever been smoke more than 100 cigarette? ① Yes ② No

Do you smoke these days? ① Every day ② Some times ③ No

**Alcohol consumption** How many times do you drink alcohol? ① less than 1 time per month ② 1 time per month ③ 2-4 times per month ④ 2-3 times a week ⑤ 4 or more times a week ⑥ Never

**Sleep** How many times do you sleep usually? ___ hours

**Stress** What is your stress level in everyday life? ① severe ② moderate ③ some ④ no

**The sedentary time** How much time did you spend per day sitting to watch TV, play games, use the internet, or do other things, during your leisure time on weekdays during the past week? ① < 1 hour ② ≥ 1 hour, < 2 hours ③ ≥ 2 hours, < 3 hours ④ ≥ 3 hours, < 4 hours ⑤ ≥ 4 hours

**Hypertension** Have you ever been diagnosed as hypertension by a medical doctor? ① Yes ② No

**Diabetes mellitus** Have you ever been diagnosed as diabetes mellitus by a medical doctor? ① Yes ② No

**Hyperlipidemia** Have you ever been diagnosed as hyperlipidemia by a medical doctor? ① Yes ② No
