# Supplementary material for: Leisure sedentary time is differentially associated with hypertension, diabetes mellitus, and hyperlipidemia depending on occupation
Source: BMC Public Health. 2017 Mar 23;17:278. doi: 10.1186/s12889-017-4192-0 (PMC5364658; doi:10.1186/s12889-017-4192-0)
Supplement: Supplementary file 2 — Odds ratios of sedentary time for hypertension, diabetes mellitus, and hyperlipidemia using multiple logistic regression analyses with complex sampling. In other statistical model, the odd ratios of sedentary time were described. (DOCX 20 kb) [file 12889_2017_4192_MOESM2_ESM.docx]

Supplement 2. Odds ratios of sedentary time for hypertension, diabetes mellitus, and hyperlipidemia using multiple logistic regression analyses with complex sampling

| Sedentary Time (h) | | | Hypertension | | Diabetes Mellitus | | Hyperlipidemia | |
| --- | --- | --- | --- | --- | --- | --- | --- | --- |
|  | |  | AOR (95% CI) | P Value | AOR (95% CI) | P Value | AOR (95% CI) | P Value |
| Multiple Regression (model 1) | | |  | <0.001* |  | <0.001* |  | <0.001* |
|  | <1h | | 1 |  | 1 |  | 1 |  |
|  | ≥1h, <2h | | 1.01(0.96-1.06) |  | 1.00 (0.92-1.08) |  | 0.96 (0.91-1.02) |  |
|  | ≥2h, <3h | | 1.07(1.02-1.13) |  | 1.09 (1.01-1.18) |  | 1.02 (0.97-1.08) |  |
|  | ≥3h, <4h | | 1.15(1.08-1.22) |  | 1.30 (1.18-1.43) |  | 1.16 (1.08-1.24) |  |
|  | ≥4h | | 1.33(1.26-1.41) |  | 1.60 (1.47-1.75) |  | 1.29 (1.21-1.37) |  |
| Multiple Regression  (model 2) | | |  | <0.001* |  | <0.001* |  | <0.001* |
|  | <1h | | 1 |  | 1 |  | 1 |  |
|  | ≥1h, <2h | | 1.02 (0.97-1.07) |  | 1.02 (0.94-1.11) |  | 0.98 (0.93-1.03) |  |
|  | ≥2h, <3h | | 1.05 (1.00-1.11) |  | 1.09 (1.00-1.18) |  | 1.04 (0.98-1.10) |  |
|  | ≥3h, <4h | | 1.09 (1.03-1.17) |  | 1.23 (1.12-1.36) |  | 1.15 (1.08-1.23) |  |
|  | ≥4h | | 1.22 (1.15-1.30) |  | 1.50 (1.28-1.53) |  | 1.24 (1.17-1.33) |  |

*Significance at P < 0.05.

Independent factors of model 1: Age, sex, and leisure sedentary time.

Independent factors of model 2: Age, sex, income, obesity, education, alcohol, smoking, stress, sleep, and leisure sedentary time.
