# Supplementary material for: Leisure sedentary time is differentially associated with hypertension, diabetes mellitus, and hyperlipidemia depending on occupation
Source: BMC Public Health. 2017 Mar 23;17:278. doi: 10.1186/s12889-017-4192-0 (PMC5364658; doi:10.1186/s12889-017-4192-0)
Supplement: Supplementary file 3 — Subgroup analysis of adjusted odds ratios of sedentary time for hypertension, diabetes mellitus, and hyperlipidemia according to the occupation groups in female using multiple logistic regression analyses with complex sampling. This data shows the odd ratios of female groups. (DOCX 21 kb) [file 12889_2017_4192_MOESM3_ESM.docx]

Supplement 3. Subgroup analysis of adjusted odds ratios of sedentary time for hypertension, diabetes mellitus, and hyperlipidemia according to the occupation groups in female using multiple logistic regression analyses with complex sampling

|  | | Hypertension | | Diabetes Mellitus | | Hyperlipidemia | |
| --- | --- | --- | --- | --- | --- | --- | --- |
| Sedentary Time (h) | | AOR (95% CI) | P Value | AOR (95% CI) | P Value | AOR (95% CI) | P Value |
| Employed  (n = 78,188) | |  | 0.484 |  | 0.032* |  | 0.021* |
|  | <1h | 1 |  | 1 |  | 1 |  |
|  | ≥1h, <2h | 1.06 (0.97-1.16) |  | 1.11 (0.94-1.29) |  | 0.99 (0.90-1.09) |  |
|  | ≥2h, <3h | 1.02 (0.92-1.13) |  | 1.10 (0.93-1.30) |  | 1.04 (0.94-1.15) |  |
|  | ≥3h, <4h | 1.11 (0.98-1.26) |  | 1.15 (0.94-1.42) |  | 1.09 (0.96-1.24) |  |
|  | ≥4h | 1.05 (0.92-1.20) |  | 1.39 (1.14-1.70) |  | 1.21 (1.06-1.38) |  |
| Unemployed  (n = 49,339) | |  | < 0.001* |  | <0.001* |  | <0.001* |
|  | <1h | 1 |  | 1 |  | 1 |  |
|  | ≥1h, <2h | 1.09 (0.95-1.24) |  | 1.25 (1.02-1.53) |  | 0.98 (0.85-1.13) |  |
|  | ≥2h, <3h | 1.18 (1.03-1.35) |  | 1.27 (1.04-1.56) |  | 1.09 (0.94-1.26) |  |
|  | ≥3h, <4h | 1.15 (0.99-1.33) |  | 1.38 (1.10-1.72) |  | 1.27 (1.08-1.48) |  |
|  | ≥4h | 1.27 (1.11-1.46) |  | 1.48 (1.21-1.81) |  | 1.25 (1.08-1.45) |  |

*Significance at P < 0.05

Independent factors of multiple regression: Age, income, obesity, education, alcohol, smoking, stress, physical activity, sleep, and leisure sedentary time.
